# Modeling endosymbioses: Insights and hypotheses from theoretical approaches

**DOI:** 10.1371/journal.pbio.3002583

**Published:** 2024-04-10

**Authors:** Lucas Santana Souza, Josephine Solowiej-Wedderburn, Adriano Bonforti, Eric Libby

**Affiliations:** 1 Department of Mathematics and Mathematical Statistics, Umeå University, Umeå, Sweden; 2 Integrated Science Lab, Umeå University, Umeå, Sweden; 3 Department of Ecology and Environmental Science, Umeå University, Umeå, Sweden; 4 Umeå Marine Sciences Centre, Umeå University, Norrbyn, Sweden; University of Oxford, UNITED KINGDOM

## Abstract

Endosymbiotic relationships are pervasive across diverse taxa of life, offering key avenues for eco-evolutionary dynamics. Although a variety of experimental and empirical frameworks have shed light on critical aspects of endosymbiosis, theoretical frameworks (mathematical models) are especially well-suited for certain tasks. Mathematical models can integrate multiple factors to determine the net outcome of endosymbiotic relationships, identify broad patterns that connect endosymbioses with other systems, simplify biological complexity, generate hypotheses for underlying mechanisms, evaluate different hypotheses, identify constraints that limit certain biological interactions, and open new lines of inquiry. This Essay highlights the utility of mathematical models in endosymbiosis research, particularly in generating relevant hypotheses. Despite their limitations, mathematical models can be used to address known unknowns and discover unknown unknowns.

## Abstract 2

Endosymbiotic relationships are pervasive across diverse taxa of life, offering key insights into eco-evolutionary dynamics. This Essay highlights the utility of mathematical models in endosymbiosis research, particularly in generating novel hypotheses, arguing that they serve as a useful complement to empirical approaches.

## Introduction

Endosymbioses are important drivers of eco-evolutionary dynamics that have the potential to forge entirely new kinds of individuals [1–4]. For example, the emergence of eukaryotes is intertwined with an endosymbiosis that would eventually evolve into mitochondria. Since gaining mitochondria, eukaryotes have demonstrated a great facility for establishing additional endosymbioses [5–7] and some may depend on their endosymbiotic associations for survival (e.g., aphids with Buchnera [8] and nemotodes with Wolbachia [9]). Yet, even in associations with incredible functional integration, endosymbioses are dynamic: relationships change, partners are abandoned or swapped, and new types or levels of interactions emerge. The signatures of these past associations can persist in genomes and may influence future relationships [10,11]. It is the complexity, ubiquity, and significance of endosymbioses that make them fascinating subjects to study.

Given the pervasiveness of endosymbioses, it is perhaps not surprising that there is a wide range of empirical and experimental systems that vary across environments and taxa. If we consider systems organized by the size of the host cell, we can start with prokaryote hosts. Endosymbioses among prokaryotes are extremely rare and so, as a proxy, most attention has been dedicated to the origin and evolution of the mitochondria within protoeukaryotes [12,13]. Within eukaryotes, there are many unicellular hosts with prokaryote endosymbionts (e.g., protists [14]), and some have been used as experimental models of endosymbioses, such as *Paramecium bursaria* [15]. Even in these relatively small organisms, there can be multiple types of endosymbionts and associated microbial communities. Similarly, large-scale organisms such as multicellular eukaryotes can have many endosymbionts and even endosymbionts that have their own endosymbionts [16]. Experimental models of these larger organisms often purposely select for relationships based on their ease of study and control, for example, the endosymbiosis between the Hawaiian bobtail squid *Euprymna scolopes* and *Vibrio fischeri* [17]. There are also many systems that lie somewhere on the spectrum of endosymbioses, such as plasmids and microbiomes that overlap with other fields of research including virology and microbial ecology. With all of this variation, it can be difficult to tease apart general features from organismal idiosyncrasies.

Mathematical modeling can serve as a useful complement to empirical techniques by allowing researchers to better understand their systems and place them in a wider context. By making the theory explicit, they can also serve as a universal language for collaboration, enabling research to be integrated from a range of disciplines [18,19] (see also [20,21] for how to interpret and integrate models with experiments), at times offering remarkable insights into biological systems [22] and simultaneously opening new fields of mathematics research [23]. Some recent reviews highlight the particular usefulness of mathematical models in evolutionary biology [24,25]. The first demonstrates how the rigorous logic of a mathematical formulation can identify the factors that facilitate the evolution of sexes or new species. The second outlines the insight that mathematical models can bring to the evolution of stress responses by integrating physiological mechanisms with an evolutionary optimality analysis. In this Essay, we consider the use of models to specifically address topics concerning endosymbioses and the types of hypotheses modeling can generate.

### Integration of contrasting effects

A key question in the evolution of endosymbioses concerns the nature of the relationship between a host and its endosymbiont [26–28]. If the relationship is exploitative, then a coevolutionary arms race might ensue. If, instead, the relationship is mutualistic, then tighter integration and division of labor may evolve. Determining the nature of the interaction can thus lead to different predictions concerning the evolution of the relationship.

Empirical evidence suggests that the nature of the host–endosymbiont relationship is highly dynamic, changing across environmental conditions and time [29,30]. For example, changing the intensity of light shifted whether carrying green algal endosymbionts was costly or beneficial for its host [15]. In another interesting example, a five-year coculture evolved to change the interaction between the host and endosymbiont from initially exploitative to mutualistic [31]. A major determinant of such social evolution is the overall impact of the costs and benefits associated with behaviors, which influences the strength and mode of selection [32,33]. Since costs and benefits can change across environments and time scales, it can be difficult to determine their net effect over different contexts without using quantitative approaches.

**Fig 1:**
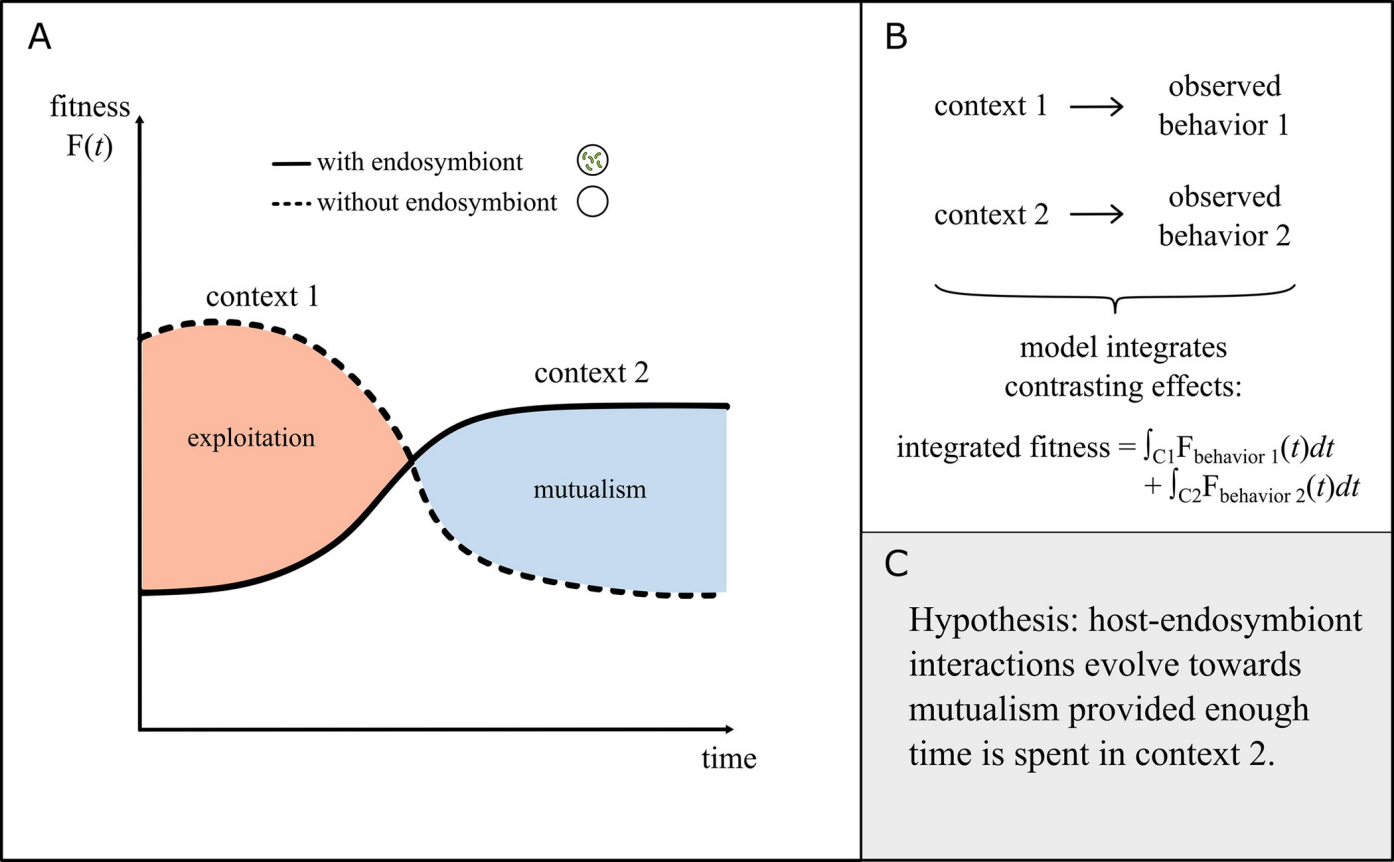
Integrating contrasting effects. (**A**) Sketch illustrating how the fitness of a host with or without endosymbionts changes in different contexts. These contexts affect whether selection favors mutualistic or exploitative relationships. Integrating over all contexts (e.g., the whole life cycle) gives a perspective on the overall behavior. (**B**) Schematic showing how 2 contrasting behaviors might be observed under different contexts. Integrating over the whole time period identifies the net outcome. (**C**) An example hypothesis generated for an endosymbiosis that appears to exhibit 2 contrasting behaviors—exploitation and mutualism—in different contexts.

Mathematical models can reveal surprising biological patterns when the interaction between costs and benefits is dynamic. For example, while vertical transmission was thought to be the primary way to reduce virulence in viruses, a mathematical model showed the opposite (i.e., that horizontal transmission also selects for lower virulence in viruses) [34]. Within the context of endosymbiosis research, an experiment showed that the *Dictyostelium discoideum* amoebae carrying *Paraburkholderia* bacteria intracellularly experienced a benefit in nutrient-poor environments but not in nutrient-rich ones [29,35]. However, it was unclear whether carrying an endosymbiont would be beneficial in environments that switched between nutrient-rich and nutrient-poor conditions. Since the environments fluctuate, it might be reasonable to hypothesize that bet-hedging strategies would occur so that some, but not all, members of the population would carry endosymbionts. A mathematical model integrating the various costs and benefits tested this hypothesis and found that, contrary to expectation, bet-hedging strategies were rarely selected [29]. These examples highlight how mathematical models that integrate across different contexts can offer new insights into the dynamic nature of endosymbiosis interactions (Fig 1).

### Revealing cross-system patterns

By employing abstractions to concentrate on processes and interactions, mathematical models effectively highlight connections between different fields and identify general themes. The typical modeling procedure entails abstracting a biological system, analyzing the model, and subsequently deducing implications for the biological system. This final stage offers an opportunity to generalize beyond the initial system. In modeling an endosymbiosis, the extent of these generalizations typically depends on how restrictive a model is. For example, a theoretical model was designed to investigate if a microbiome could induce cooperative behavior in its host, with minimal restrictions on the microbiome itself [36]. The degree of abstraction present in this model allows its predictions to be applied to a broader range of entities that transmit between organisms, including plasmids, viruses, and multicellular symbionts.

Theoretical approaches can also identify fields that may benefit from exchange. For example, endosymbioses and certain microbial communities share similar patterns of division of labor, with both undergoing significant gene loss and evolving obligatory dependencies [37,38]. A challenge arising from such division of labor is coordination (i.e., who does what and when). If a host relies on its endosymbiont to produce energy, but sufficient energy is not provided, then the system can collapse. Understanding how coordination evolves is relevant to many areas of research in biology, including endosymbiosis, microbiomes, microbial community assembly, multicellularity, and mutualisms. These research areas all explore the ways in which selection acting on a system as a whole can lead to improved system performance, usually through some measure of fitness or function. Exchanges between these areas may help elucidate important mechanisms and common features of how multispecies systems can coordinate division of labor.

**Fig 2:**
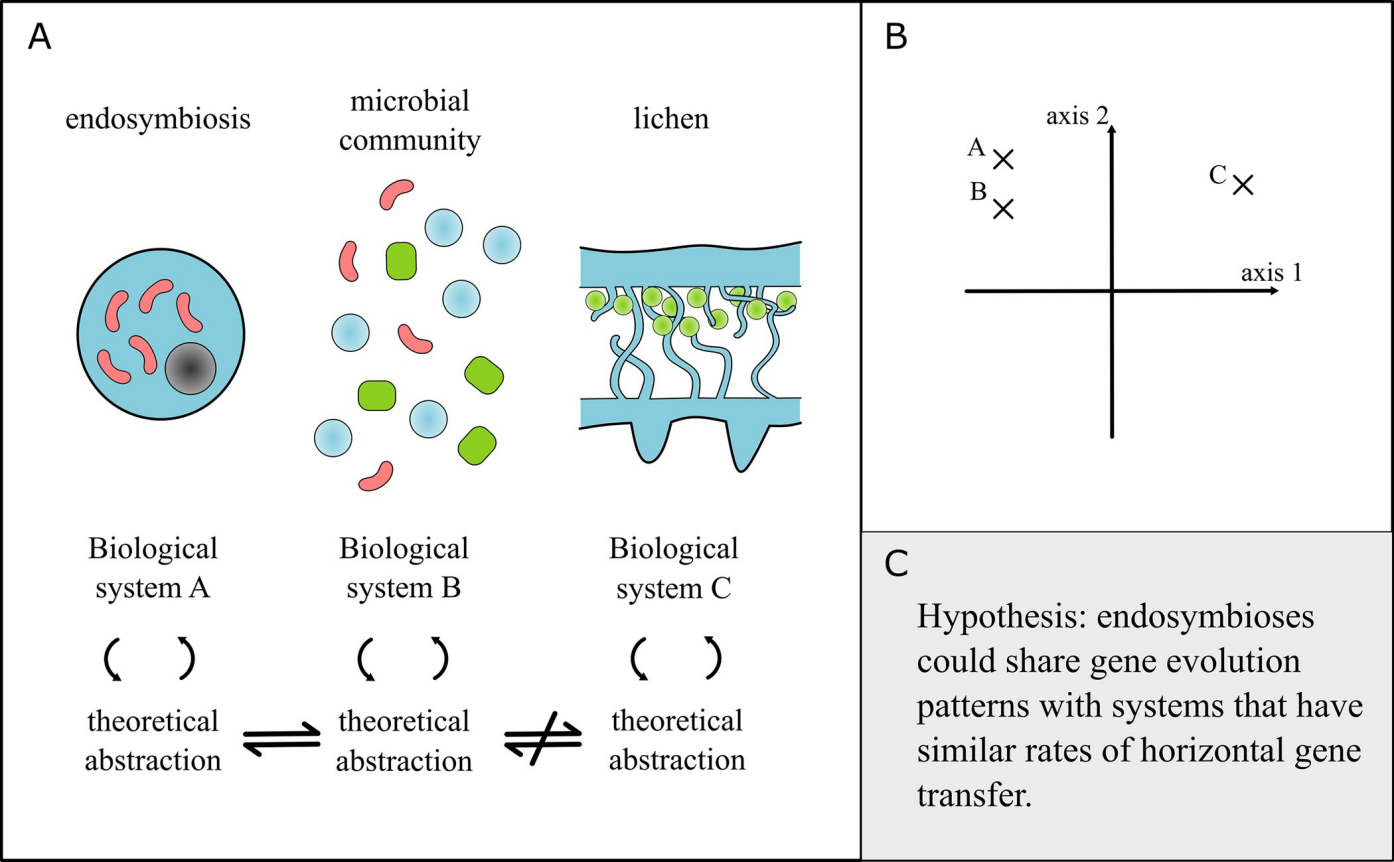
Broad generalizations. (**A**) Sketch illustrating how theoretical abstraction can be used to compare between different biological systems. For example, this can help to identify similarities in division of labor between some endosymbioses and microbial communities. Theoretical abstractions also highlight distinctions between systems (e.g., lichens have a lower frequency of horizontal gene transfer [43] than microbial communities). (**B**) Schematic showing how different axes can be used to separate biological systems. Here, systems A, B, and C could display similar dynamics along axis 2, but system C might be incomparable with A and B along axis 1. Moving along the axes could be interpreted in 2 ways depending on the scenario: (i) changing a parameter value, such as rate of horizontal gene transfer, shifts the system into a different regime where categorically different behaviors are observed; and (ii) entirely different models, such as mode of transfer, are required. (**C**) An example hypothesis generated for endosymbioses that broadly share similar rates of horizontal gene transfer.

Another productive outcome of generalizing a mathematical model lies in exploring what happens when it does not apply to another system. Such failures in generalization can reveal useful distinctions that serve to organize scientific fields (Fig 2). For example, a model for insect–aphid symbiosis may not apply to plasmids because of the different frequencies in vertical versus horizontal transmission; this would indicate that transmission mode may be a useful axis to draw distinctions between endosymbioses. Indeed, a theoretical framework for endosymbioses organized them by the mode of transmission of endosymbionts in order to place them in the broader context of major transitions theory [2]. The value of such organization frameworks is 3-fold. First, they group systems together into categories that share similar abstractions and models, where results from one system can inform predictions about another. Second, they identify areas where empirical systems are missing, which indicates either that model systems should be developed or some constraints prevent these systems from occurring. Third, they can offer predictions on how categories evolve or transform into others (e.g., how endosymbioses may evolve into integrated units of selection/multispecies individuals) [2,39–42]. Overall, these generalizations provide a common language to explore questions spanning the spectrum of different endosymbioses.

### Evaluating mechanisms

**Fig 3:**
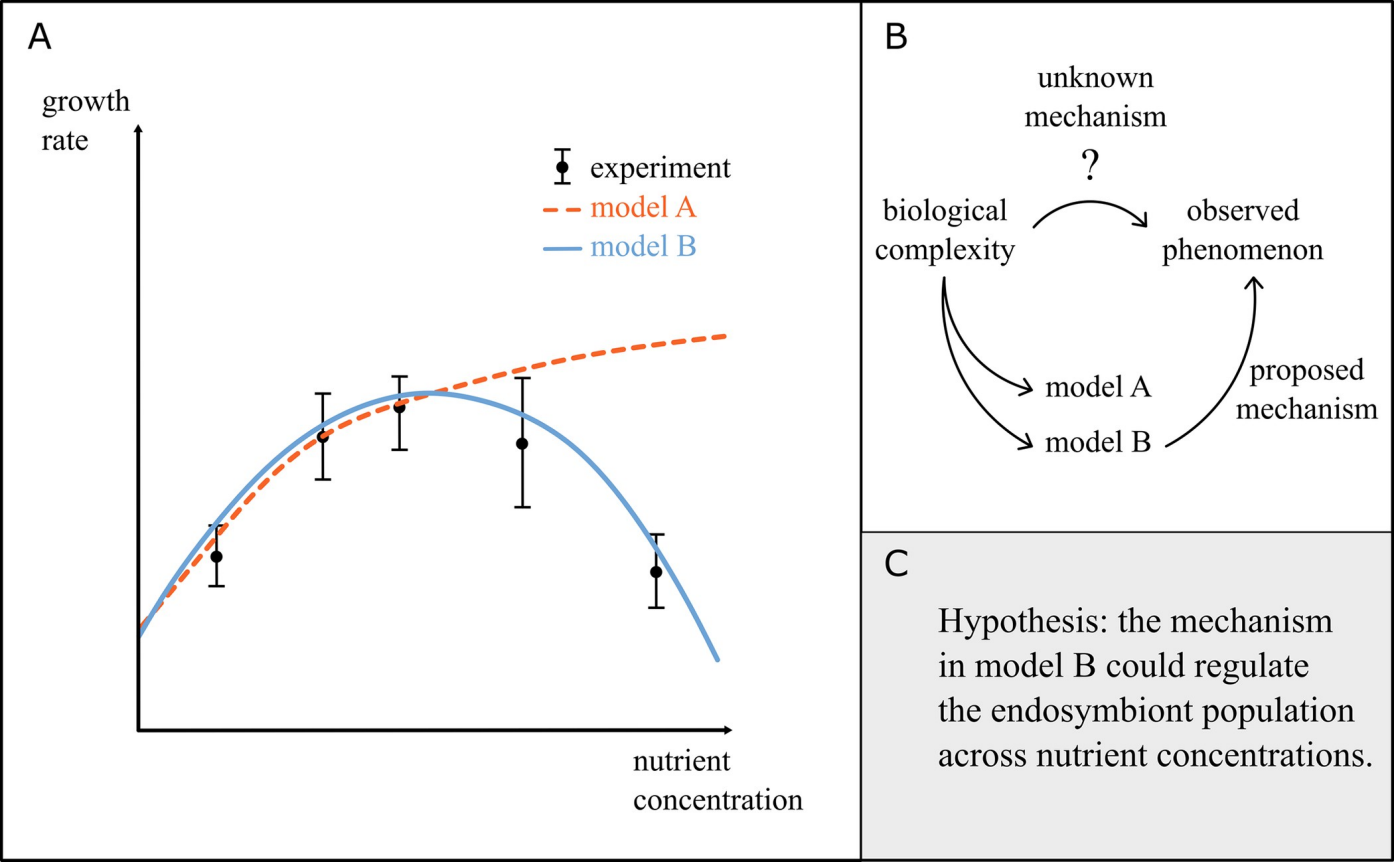
Identifying potential mechanisms. (**A**) Sketch illustrating 2 models for the growth of an endosymbiont under different nutrient concentrations with experimental results (inspired by [15]); model B is a better fit for the data. (**B**) Schematic showing how models can propose mechanisms for an observed phenomenon when the full details of the underlying mechanism are unknown. More than 1 model system can be explored to see when the outputs match the observations. (**C**) An example hypothesis generated for the regulation of endosymbionts where the primary mechanism is currently unknown.

Biological systems are composed of a tangled web of interconnected components, which makes it difficult to identify the primary mechanisms responsible for a given phenomenon or behavior. To make matters worse, it is often unclear whether a crucial piece of information is missing. In such situations mathematical models can be extremely useful, providing a way to evaluate whether a set of components are sufficient to “explain” the phenomenon (Fig 3). An example in endosymbiosis research is how hosts and endosymbionts navigate their relationships to maintain stability. Relationships between hosts and their endosymbionts are often regulated by a vast repertoire of chemical exchanges and physical interactions. Developing mathematical models for specific subsets of these exchanges and interactions can provide hypotheses for the primary mechanisms driving them. These hypotheses can then be experimentally explored as part of a collaborative experimentation–modeling process to identify underlying mechanisms.

When we lack empirical observations or a complete underlying molecular description, mathematical models can be used to assess whether a proposed mechanism is sufficient. An example of this in the endosymbiosis literature is a mathematical model that was developed to explain how the unicellular ciliate *P*. *bursaria* maintains a stable population size of its algal endosymbiont [44]. Since many of the molecular details of the underlying mechanism were missing, there were competing hypotheses for how stability could be maintained. In a combination of experiments and mathematical modeling, the authors demonstrated that a difference in the nutritional growth requirements of the host and endosymbiont was sufficient to replicate empirical observations and give rise to a stable maintenance of the population. The simple mechanism uncovered by this paper has additional properties with further implications (i.e., the mechanism functions across different growth conditions and does not require extensive coordination, which suggests it could evolve readily in different endosymbioses).

In addition to hypotheses for specific empirical systems, mathematical models can offer hypotheses concerning as yet unobserved phenomena and where to look for them. A particularly pertinent question is how host–endosymbiont relationships can become more intertwined, leading to a greater reliance on vertical rather than horizontal transmission. One model tackling this issue revealed that self-regulation would only evolve if the benefits of the relationship are sufficiently high for both the host and endosymbiont [45]. Analysis of the model also provided an explanation as to why benefits to both were necessary: when the benefit to hosts is large, then those without endosymbionts are outcompeted and lost in the population. As a result, endosymbionts effectively lose the horizontal route of transmission and stay with their hosts longer, leading them to evolve regulation of their own population size. A second theoretical study examined the costs endosymbionts face in finding new hosts and how this implicates host–endosymbiont relationship dynamics [46]. This model showed that it is more likely for endosymbionts to lose the ability to live freely and reproduce independently when host encounters are both rare and costly. Together, these results not only show when and why it might be possible for endosymbionts to control their own population, but also provide some prediction of what kinds of endosymbioses might foster this behavior.

### Explore the unknown

**Fig 4:**
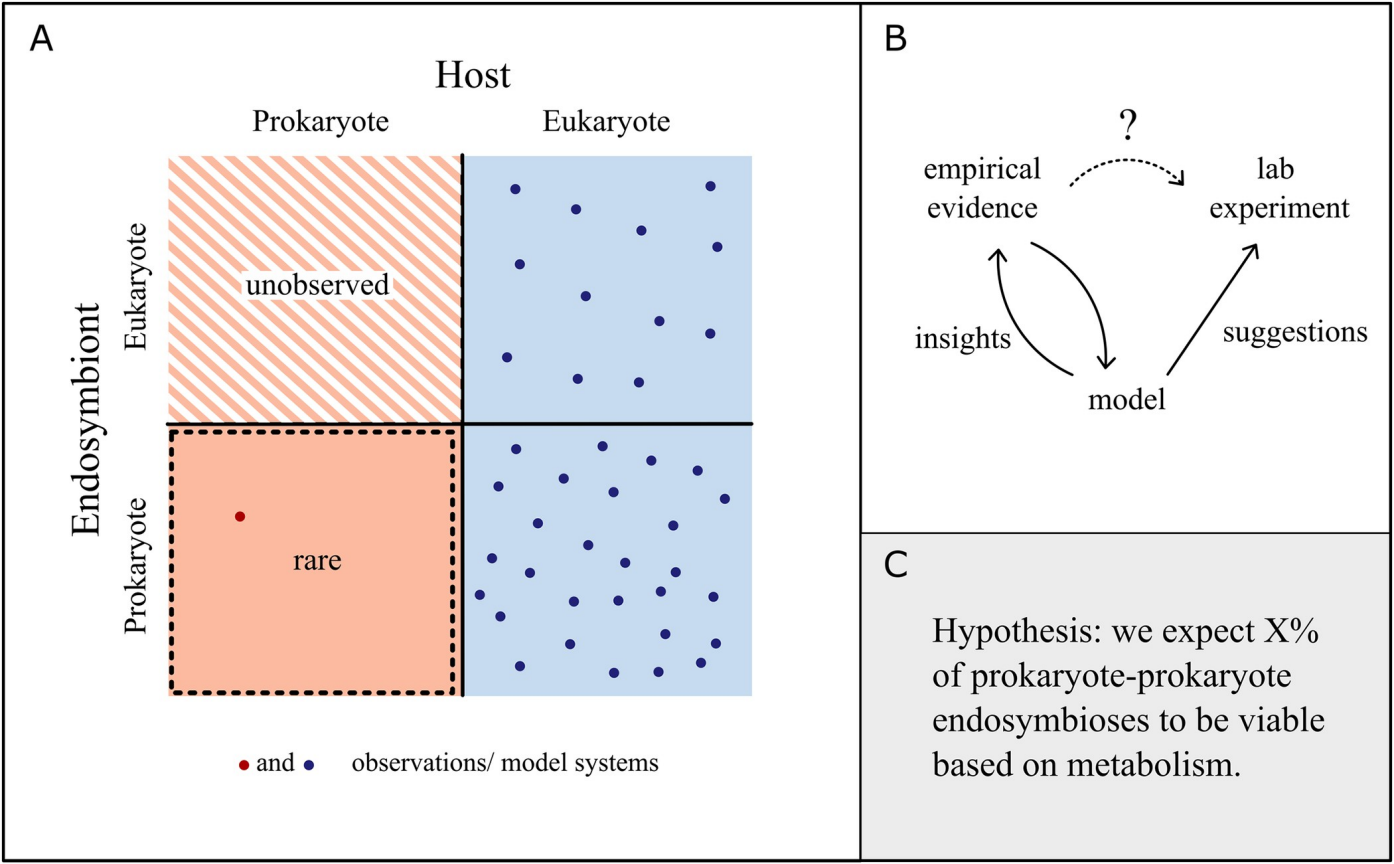
Exploring the unknown. (**A**) Several examples of endosymbioses with a eukaryote are known and well studied, but prokaryote hosts are lacking. Prokaryote hosts with eukaryote endosymbionts have not been observed and might be unfeasible due to space limitations. Prokaryote–prokaryote endosymbioses are extremely rarely observed. These endosymbioses can be explored with theoretical models, for example, to estimate the proportion of prokaryote–prokaryote pairs that could form viable endosymbioses based on metabolism (as in [51]). (**B**) Schematic showing how modeling can be used to give insights for empirical systems where lab experiments have yet to be devised. The model can suggest potential experimental systems, for example, by identifying species that have a high propensity to become hosts and endosymbionts. (**C**) An example hypothesis generated for prokaryote–prokaryote endosymbioses where experimental systems are currently unknown.

Within the field of endosymbiosis, there is a glaring absence of endosymbioses featuring prokaryote hosts. There are only a few observed examples besides possibly the endosymbiosis that would give rise to the mitochondria [16,47,48]. This rarity is surprising when compared to the frequency of eukaryote endosymbioses and when considering the diversity and abundance of prokaryotes. There have been different qualitative arguments as to why prokaryote endosymbioses may be rare [13,49,50], but since we lack experimental systems, it is difficult to identify what factors are responsible for the rarity. Here, mathematical models can be particularly helpful in evaluating and comparing hypotheses for rarity and determining what constraints limit the successful establishment of prokaryotic endosymbioses (Fig 4).

An example of this use of mathematical modeling can be seen in a recent study that considered the role of metabolic compatibility in prokaryote endosymbioses [51]. Prior to this work, some had argued that prokaryote endosymbioses may not be viable without a prior history of coevolution (see [13] for an excellent review). For example, if an endosymbiont needs some essential compound to grow but the host cell lacks a way of transporting this compound into the cell, then the endosymbiosis would not be viable. However, it is unclear how often this type of scenario would be expected to occur in pairs of prokaryotes (i.e., to what extent is this actually a driver of the rarity). Mathematical models can be used to estimate this and thus offer a null model prediction that can later be refined or assessed by incorporating other, possibly empirical, data. In this particular case, bacterial metabolic models were randomly paired as in silico endosymbioses. Over half of the pairs tested were metabolically viable, though very rarely did they have higher fitness (growth rate) than their ancestors. Without empirical systems, it can be difficult to validate these quantitative models or assess their accuracy, yet they provide a null prediction that can be used to set expectations and compare with future findings.

### New lines of inquiry

Mathematical models can be used to establish new lines of inquiry both in terms of scale and direction. These uses of modeling are well-illustrated in studies of corals and their dinoflagellate endosymbionts, which provide an exceptional system for studying endosymbiosis because there is abundant data in terms of spatial variation of coral communities, as well as a rich historical record of environmental conditions and coral growth. This data has led to the development of intricate models that explicitly define the many mechanisms and processes that affect individual organism behaviors and their interactions with the environment. In particular, agent-based models excel in capturing heterogeneity and spatial interactions among individuals and other components of the system, crucial for accurately modeling complex ecosystems like coral reefs [52–54]. Because these models are very complex, they are often calibrated and validated by comparing their hindcast predictions with historical data, thereby improving their reliability for future projections about coral health in response to a changing climate [55]. Given the relevance of such historic data, recent efforts have focused on extending the temporal timescale further back to procure more robust data [56], creating lines of inquiry for endosymbiosis that consider timescales beyond human lifetimes.

**Fig 5:**
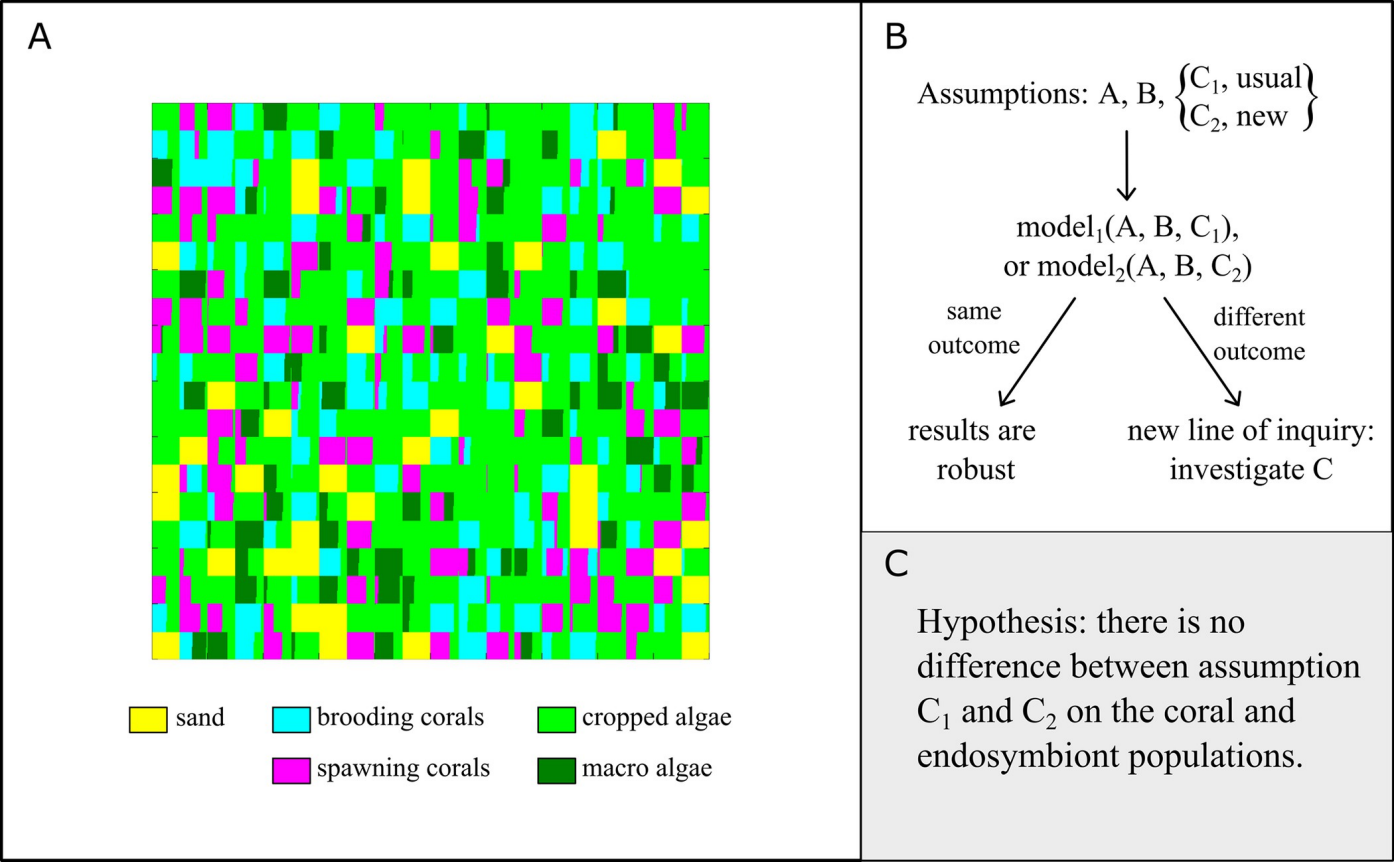
New lines of inquiry. (**A**) A representative output from agent-based model simulations of a coral ecosystem, adapted from [53]. Each grid square’s color represents the relative proportions of sand, various coral species, and algal types. To enhance this model by incorporating the impact of endosymbionts on coral growth rates and mortality during stressful events, such as heatwaves [54], assumptions are required regarding the interactions between corals and their endosymbiotic algae (for example, how corals may alter their internal symbiont composition in response to such events). (**B**) Schematic illustrating the assumptions necessary for constructing a model. Two potential assumptions for component C are presented. By running simulations with both assumptions, we can test the robustness of the results; identical outcomes suggest that the model’s results are not sensitive to the assumptions about C. Conversely, divergent outcomes indicate that further investigation into assumption C could lead to new research avenues. (**C**) An example hypothesis generated for a coral endosymbiosis where different assumptions can be made.

Both creating a mathematical model and analyzing it can uncover hidden assumptions and reveal new directions to explore (Fig 5). The process of writing down a model, such as an agent-based model, forces one to make many explicit decisions on how agents behave and within what space they interact. Reflecting on these decisions can identify unknown details and outstanding questions. For example, in [54], the authors extended a spatially explicit agent-based model for corals [53] to assess the long-term benefits of switching from thermally sensitive to thermally tolerant symbionts after heat waves. In developing the model, the authors faced the decision of whether to include symbiont reversal to the original composition after some time or to keep the switch from sensitive to tolerant symbionts unidirectional. Including symbiont reversal would require also knowing the relationship between the dynamics of symbiont switching and coral growth. The authors concluded that the empirical data to characterize this was insufficient at the time and assumed the switch from sensitive to tolerant symbionts was unidirectional. This is a clear example of a situation in which the implementation of a model forces the authors to make certain assumptions explicit (the reversibility of symbiont switching) and points to new lines of inquiry (how to characterize the switching dynamics).

In some cases, there is not enough evidence to distinguish between competing assumptions in a model. Experiments may help determine which assumption is better supported, but conducting experiments for every modeling assumption is not cost-effective. By analyzing a model using each assumption, we can explore whether the competing assumptions actually lead to different outcomes. To see how this works, we consider the earlier case of the assumption concerning symbiont switching. Models could be run with different assumed relationships between coral growth and symbiont switching dynamics as a kind of sensitivity analysis. If a wide swath of dynamic relationships produce the same behavior, then this would suggest that while the exact relationship is unknown, it may not be as important to investigate as other parameters. If instead different dynamic relationships produce different behaviors, then this would indicate that this parameter should be investigated. Since computations tend to be cheaper and faster than experiments, this methodology can determine which of the unknowns in a model are the most important to learn.

### Frameworks for integration

With the variety of empirical systems and theoretical approaches available, we also need ways of combining them into common frameworks to address central questions on endosymbioses. This comes with challenges, such as simplifying complex problems, ensuring realistic results, identifying influential variables, and requiring iterative refinement. Although in principle there are many ways of doing this (e.g., Bayesian models), an interesting way that may be relevant for questions in endosymbiosis research is the use of Fermi estimates. Fermi estimates simplify complex problems by dividing them into smaller, more manageable parts and using educated guesses. Possibly the most famous example of a Fermi estimate is the Drake equation, which tries to estimate the number of radio-communicative civilizations in the Milky Way [57,58]. This involves estimating factors such as the rate of star formation, the fraction of those stars with planets, and the likelihood of life developing intelligence.

To see how Fermi estimates may be useful in endosymbiosis research, one could consider the question of why prokaryote endosymbioses are rare. This question could be addressed by estimating the likelihood of establishing a prokaryote endosymbiosis, which in turn could be broken down into the following terms: the total number of interactions between different prokaryotic species; the fraction of those encounters that lead to one cell getting inside another; the fraction of those newly created endosymbioses that can reproduce; and the fraction of those viable endosymbioses that persist long enough to fix in a population. Though we might not know the precise value of any of these terms, we can make educated guesses for some by using existing empirical data. For example, we can estimate the first term by multiplying the average number of interactions per species [59] with the number of prokaryote species [60]. Where data is unavailable, theoretical approaches can provide approximations for some of the gaps; for example, metabolic models have been used to estimate the fraction of newly created endosymbioses that are viable [51]. Of course, this approach to estimation raises all sorts of possible issues and omissions; however, addressing these factors and determining how to incorporate them is part of the value of such a formulation.

## Conclusions

In this Essay, we have explored how mathematical models can serve as useful tools in endosymbiosis research, showcasing the types of hypotheses mathematical models can generate and how they can be used to complement empirical approaches. We purposely selected examples representing a diverse set of models and model systems to highlight the breadth of possibilities in terms of the utility and application of mathematical models. To get a sense of the types of questions within endosymbiosis research that mathematical modeling may be particularly well-suited to address, we outline some key questions in Box 1. Many of the modeling examples considered in this Essay address one of these questions (e.g., coral endosymbiosis models often address the first question, concerning the effects of environmental conditions). Yet, there are some questions that have been relatively unexplored. For example, the third question poses how an endosymbiosis may respond to an additional element such as another endosymbiont or a virus. This question is relevant in terms of plastid acquisition, whereby eukaryotes have gained additional endosymbionts following the mitochondria. Regardless of the extent to which these questions have been considered, the tremendous diversity of endosymbioses means there is plenty of room for exploration, both building on existing models and in new directions.

**Box 1. Topics in endosymbiosis fit for mathematical modeling**

1. **Dynamic environments and evolving relationships:** Do changing environmental conditions alter the costs/benefits of endosymbioses and their evolution along the mutualism–exploitation spectrum?
2. **Coordination of reproduction:** How do endosymbioses ensure consistent, synchronized reproduction of host and endosymbionts in the face of possible conflicts over resource use and selection to maximize growth rate?
3. **Third party influence:** How do endosymbioses respond to the introduction of a third species such as another endosymbiont or a virus?
4. **Division of labor:** In what ways can endosymbioses partition labor (e.g., energy transformation or metabolism) to gain sustainable, synergistic benefits?
5. **Comparative effects of spatial organization:** When does the specific spatial arrangement of endosymbioses offer different outcomes than symbioses between partners that are not arranged in a nested architecture?
6. **Harmonious pairings:** Are there certain types of species that are more likely to produce a successful endosymbiosis than others?
7. **Long-term horizon:** What factors influence whether an endosymbiont remains a long-term partner, evolves into an organelle, or deteriorates until lost or replaced?

Certainly mathematical models have their limitations, and for many relevant questions in endosymbioses it is better to interrogate an experimental system or empirical data. But there are some tasks for which mathematical models are particularly well suited. Moreover, the act of producing a model can be informative in and of itself, because it requires explicitly formulating assumptions and identifying what necessary information is missing. For now, there is a distinction in many fields of biology, such as endosymbiosis, between modelers and empiricists/experimentalists. They often collaborate, but owing to their different methodologies and academic backgrounds, they sometimes occupy different spaces within the same field. In this way, modelers exist as a type of endosymbiont within their host biological fields, seeking inspiration and exciting questions from the complexities and mysteries of biological systems. While there are costs to having modelers, hopefully the net exchange is positive, with modelers providing useful generalizations, identifying key mechanisms, and offering new insight and lines of inquiry.
